# Physics-Informed Generative Adversarial Network for Synthesis of Nonuniform Antenna Arrays with Mutual Coupling

**DOI:** 10.3390/mi17070788

**Published:** 2026-06-28

**Authors:** Li Zhang, Yiping Liu, Jie Chen, Yanshuo Shen

**Affiliations:** National Key Laboratory of Radar Detection and Sensing, Xidian University, Xi’an 710071, China; 25021211562@stu.xidian.edu.cn (Y.L.); 23021211958@stu.xidian.edu.cn (J.C.); 25021211779@stu.xidian.edu.cn (Y.S.)

**Keywords:** array synthesis, nonuniform array, mutual coupling, active element pattern (AEP), physics-informed neural network, generative adversarial network (GAN)

## Abstract

This work presents an unsupervised machine learning approach for the synthesis of nonuniform antenna arrays with consideration of mutual coupling effects. By integrating physical array synthesis formulas into the loss function, the physics-informed generative adversarial network (PI-GAN) is adopted to generate candidate designs of nonuniform antenna arrays. The generator produces the array geometric layouts and complex excitation distributions, and the discriminator assesses the fidelity of the radiation pattern relative to the design target by utilizing adversarial training with physics-driven pattern matching losses. With this proposed PI-GAN architecture, a deep neural network (DNN)-based active element pattern (AEP) surrogate model is embedded as a differentiable physics layer to accurately characterize the element mutual coupling, replacing time-consuming full-wave simulations. This end-to-end optimization paradigm enables an efficient global search over the non-convex solution space while ensuring physical consistency of the synthesized array. The method is validated on a 16-element linear array and a 300-element planar array, achieving lower peak sidelobe levels (PSLL), respectively. A prototype of the 16-element linear array is fabricated and measured, and the experimental results closely match the simulations, further validating the practical feasibility of the proposed PI-GAN synthesis framework.

## 1. Introduction

With the rapid evolution of next-generation wireless communications, high-resolution radars, and satellite Internet of Things, array antennas are required to achieve superior radiation performance and beam steering capability [1,2,3]. As a typical configuration with unequally spaced elements, nonuniform arrays can realize large apertures and narrow beams using fewer radiating elements. This configuration effectively reduces hardware size and system cost while maintaining excellent electrical performance and good engineering practicality [4]. In recent decades, heuristic algorithms such as genetic algorithm (GA) [5], particle swarm optimization (PSO) [6], and differential evolution (DE) [7] have been widely adopted for the optimal design of non-uniform arrays and achieved favorable results. Nevertheless, as the array scale increases dramatically, severe electromagnetic mutual coupling effects between elements and the highly non-convex optimization space have caused traditional heuristic array synthesis algorithms based on full-wave simulations to be plagued by the curse of dimensionality and unaffordable computational overhead [8].

In recent years, the integration of deep learning techniques [9] with computational electromagnetics has emerged as a promising paradigm to address these challenges. Various neural network architectures have been explored for antenna design and array synthesis. Artificial neural networks (ANNs) have been employed to establish surrogate models for rapid performance prediction [10,11], while deep neural networks (DNNs) have demonstrated the capability to directly map target radiation patterns to array excitation parameters [12,13]. To account for mutual coupling, active element pattern (AEP)-based methods have been combined with parallel neural networks to predict the radiation characteristics of individual elements within an array environment [14,15,16]. Convolutional neural networks (CNNs) have been introduced for pattern nulling and planar array synthesis by exploiting spatial features [17], and graph neural networks (GNNs) have shown promise in modeling arbitrary array topologies as graph structures [18].

Despite these advances, purely data-driven models often lack physical interpretability and may produce solutions that violate electromagnetic constraints. To address this limitation, physics-informed neural networks (PINNs) have been proposed to embed governing physical equations into the learning process [19]. In array synthesis, generative adversarial networks (GANs) have been adapted with physics-aware loss functions to achieve unsupervised learning for sparse array design [20]. Although some techniques have yielded competitive performance, several critical research gaps still exist. Most existing machine learning based approaches treat AEP prediction and array parameter optimization as separate sequential stages, which may lead to suboptimal solutions and convergence difficulties. Meanwhile, purely data-driven models lack physical interpretability and may produce electromagnetically invalid solutions. Moreover, the real radiation pattern data required for adversarial training in GAN-based methods are often generated from idealized array factor models that neglect mutual coupling effects. Therefore, there exists a need for a unified end-to-end optimization framework that can simultaneously handle linear and planar array synthesis with embedded inherent physical constraints.

In this paper, we propose a unified physics-informed generative adversarial network (PI-GAN) framework for nonuniform array synthesis that fully integrates mutual coupling characterization into the end-to-end optimization process. The key advantage of the proposed method is its use of GAN for powerful global optimization. A DNN-based active element pattern surrogate model is embedded as a differentiable physics layer to accurately characterize the element mutual coupling, replacing time-consuming full-wave simulations. By integrating physical array synthesis formulas into the loss function, PI-GAN is adopted to generate candidate designs for nonuniform antenna arrays. The generator produces the array geometric layouts and complex excitation distributions, and the discriminator assesses the fidelity of radiation pattern relative to the design target by utilizing adversarial training with physics-driven pattern matching losses. The presented optimization paradigm enables efficient global search over the non-convex solution space while ensuring the physical consistency of the synthesized array. Both the numerical examples and experimental results demonstrate the validity of the presented method. The main contributions of this work are threefold:

- A GAN framework is employed for nonuniform array optimization, enabling efficient global exploration of the high-dimensional, non-convex solution space and avoiding the premature convergence of traditional heuristic algorithms. Moreover, different from previous machine-learning-based array synthesis approaches merely for prediction, the developed framework is capable of executing practical end-to-end array optimization.
- A DNN-based AEP surrogate model is embedded as a differentiable physical layer to accurately characterize inter-element mutual coupling. This model effectively replaces repetitive and time-consuming full-wave simulations, ensuring rigorous physical consistency.
- The proposed method is demonstrated to be effective for both linear and planar arrays, with experimental validation on a fabricated prototype confirming its practical applicability.

The remaining part of this work is organized as follows: Section 2 introduces the operation principle and design of the proposed PI-GAN framework, numerical experimental results and discussion are presented in Section 3, and conclusions are drawn in Section 4.

## 2. Operation Principle and Design

### 2.1. Active Element Pattern Prediction Model

In practical array designs, the mutual coupling between elements significantly alters the radiation characteristics of individual elements compared with their isolated counterparts. The active element pattern (AEP) of the m-th element, denoted as $F_{m}^{e}(\theta ,\phi )$, is defined as the radiation pattern when only that element is excited while all others are terminated with matched loads. The total array pattern can be expressed as the superposition of individual AEPs weighted by complex excitations [21]:

(1) $$E_{\mathrm{total}}(\theta ,\phi )=\sum_{m=1}^{N}I_{m}\cdot F_{m}^{e}(\theta ,\phi ),$$

where $I_{m}$ is the complex excitation coefficient of the m-th element, N is the total number of elements, and $F_{m}^{e}(\theta ,\phi )$ denotes the AEP of the m-th element.

**Figure 1:**
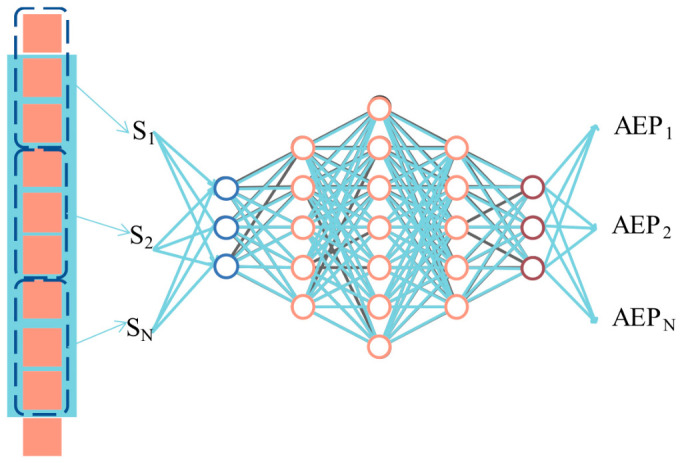
Architecture of the DNN-based AEP prediction model.

To alleviate the heavy computational burden of full-wave simulations for each candidate array configuration during optimization, we construct a DNN model to predict the AEP of each element from its local topological environment. As illustrated in Figure 1, the prediction model employs a subarray partitioning strategy for a target element, though only the distances to its nearest neighboring elements are considered as input features, based on the physical insight that mutual coupling diminishes rapidly with distance.

The input feature vector for the n-th element is defined as

(2) $$S_{n}=\left[d_{n-k},\ldots ,d_{n},d_{n+1},\ldots ,d_{n+k-1}\right],$$

where $d_{i}$ is the inter-element spacing, k is the number of adjacent elements near the target element, and zero-padding is applied for the edge elements to keep the input dimension consistent. The network outputs the magnitude and phase of the AEP at discrete angular samples, trained separately using two parallel DNNs [10] with identical architecture. The loss function is defined as the mean squared error between the predicted and simulated AEPs:

(3) $$\mathrm{Loss}=\frac{1}{N_{\theta }}\sum_{\theta \in \Theta }(f(\theta )-\hat{f}(\theta ){)}^{2},$$

where $N_{\theta }$ is the number of sampling angles, f(θ) is the simulated AEP, and $\hat{f}(\theta )$ is the predicted value.

**Figure 2:**
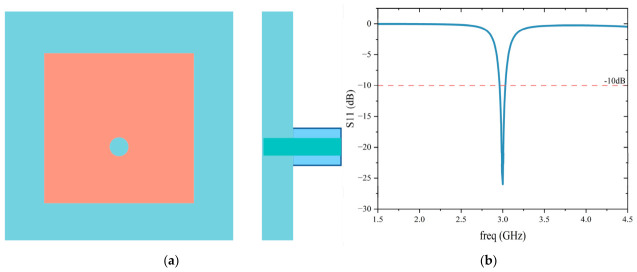
Geometry of the three GHz microstrip patch antenna elements for the linear array: (**a**) unit model, and (**b**) $S_{11}$ curve.

The antenna element employed in this work is a rectangular microstrip patch antenna operating at 3 GHz for linear arrays and 10 GHz for planar arrays. The element geometry and its $S_{11}$ performance are shown in Figure 2. The AEP model is trained with the Adam optimizer at a learning rate of $1\times 10^{-4}$, a batch size of 32, and 200 epochs. The dataset for training the AEP surrogate model is generated through full-wave electromagnetic simulations (HFSS) using the Latin Hypercube Sampling strategy. A total of 350 different array configurations are randomly generated with element spacings constrained within [0.5λ, 1.0λ], yielding 5600 AEP samples (350 configurations × 16 elements per array). The dataset is partitioned into 80% for training, 10% for validation, and 10% for testing.

The AEP prediction model consists of two parallel DNNs with identical input dimensions but different architectures. The amplitude prediction network comprises five hidden layers with neuron counts of 128, 256, 512, 256, and 128, each followed by batch normalization, ReLU activation, and dropout (rate = 0.15) applied after the first three hidden layers. The phase prediction network comprises three hidden layers with neuron counts of 128, 411, and 411, each followed by batch normalization, ReLU activation, and dropout (rate = 0.18). Both networks take the subarray spacing vector (four-dimensional input for k = 2) as an input and output 181 angular samples (0° to 180° with 1° interval). The networks are trained using the Adam optimizer with an initial learning rate of 0.001, a batch size of 64, and a maximum of 500 epochs. The learning rate is halved every 100 epochs, and early stopping with a patience of 20 epochs is employed to prevent overfitting.

**Figure 3:**
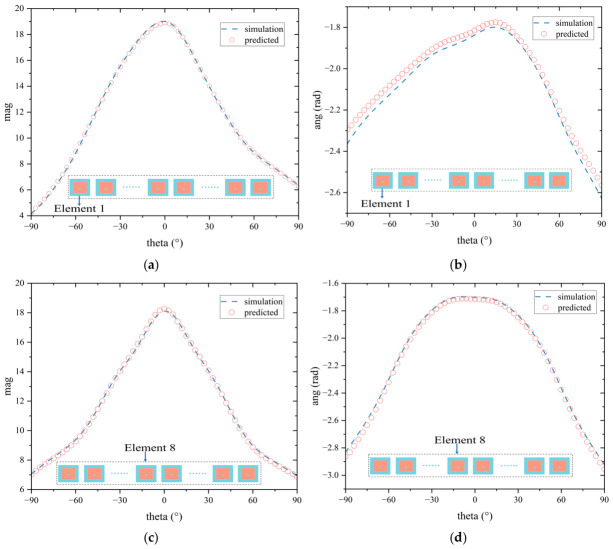
Comparison of the predicted and simulated AEP magnitudes and phases for the different element positions in a 16-element nonuniform linear array: (**a**) element 1 magnitude, (**b**) element 1 phase, (**c**) element 8 magnitude, and (**d**) element 8 phase.

Figure 3 presents a comparison between the predicted and simulated AEPs for two representative elements in a 16-element nonuniform linear array. The magnitude and phase curves show excellent agreement across the entire angular range, validating the accuracy of the surrogate model for mutual coupling effects.

Furthermore, benefiting from the two-dimensional element coupling effect, planar arrays exhibit much more complicated electromagnetic coupling characteristics. To cope with this issue, the AEP prediction strategy is improved, which introduces an effective coupling radius and adaptively builds multi-branch parallel networks based on the neighboring elements within the coupling range. The effective coupling radius is determined based on a quantitative criterion: an element is considered as a neighbor of the target element if the inter-element distance is within 1.5λ, where λ is the operating wavelength. For the microstrip patch antenna operating at 10 GHz employed in this work, this corresponds to a coupling radius of 45 mm. This threshold is determined through full-wave electromagnetic simulations, which show that the mutual coupling becomes negligible when the inter-element spacing exceeds 1.5λ. Based on this criterion, the multi-branch parallel network adaptively constructs its input features by including all neighboring elements within the effective coupling radius. The number of neighboring elements k is adaptively determined for each target element based on its local topology, and it is constrained to the range [3,12] to match the pre-trained sub-array prediction models (k = max(3, min(N_neighbors_, 12)), where N_neighbors_ is the number of elements within the coupling radius). This adaptive mechanism ensures that the dominant coupling effects are captured while maintaining computational efficiency.

### 2.2. Physics-Driven Generative Adversarial Network Framework

**Figure 4:**
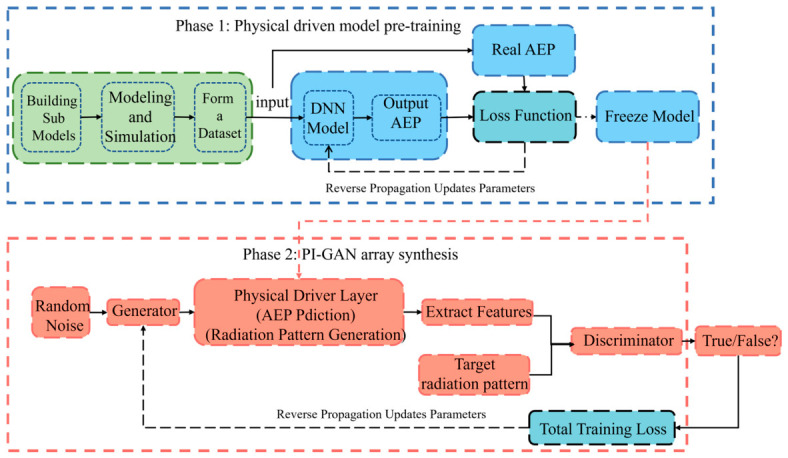
Unified architecture of the proposed PI-GAN framework for nonuniform array synthesis, applicable to both linear and planar arrays.

The core of the proposed synthesis method is a physics-informed generative adversarial network that integrates the AEP prediction surrogate model as a differentiable physics layer. The unified architecture that applies to both linear and planar arrays is illustrated in Figure 4.

In this work, the generator network consists of three fully connected layers with hidden dimensions of 128 and 256, respectively, each followed by a LeakyReLU activation function with a negative slope of 0.2. The output layer dimension equals 3N−1, corresponding to (N−1) element spacings, N excitation amplitudes, and N excitation phases. Physical constraints are enforced through activation functions. Element spacings are mapped to the range [0.5λ, 1.0λ] via a sigmoid function, excitation amplitudes are constrained to [0, 1] via sigmoid, and excitation phases are mapped to [0, 2π] via sigmoid scaling. This design ensures that all generated array layouts are physically realizable and avoid unreasonable configurations. The discriminator network also consists of three fully connected layers with hidden dimensions of 256 and 64, using LeakyReLU(0.2) activations and a dropout rate of 0.3 after the first hidden layer. The final layer employs a sigmoid activation to output a probability score between 0 and 1. The input dimension of the discriminator is 1801, corresponding to the angular sampling of the radiation pattern from 0° to 180° with a 0.1° resolution.

The generator G takes a latent vector z sampled from a standard normal distribution $P_{z}(z)=N(0,1)$ and outputs the array configuration parameters including element spacings $d_{i}$ and complex excitations $w_{i}$:

(4) $$G:G(z;\theta_{G})\to \{w_{i},d_{i}\}.$$

The physics layer is a pretrained and frozen differentiable module that computes the realistic array radiation pattern using the AEP surrogate model:

(5) $$y_{\mathrm{fake}}(\theta )=\left|\sum_{n=1}^{N}A_{n}e^{j\alpha_{n}}\cdot {\mathrm{AEP}}_{n}(\theta ,d)\cdot e^{jkx_{n}\cos\theta }\right|,$$

where $A_{n}$ and $\alpha_{n}$ are the excitation amplitude and phase, respectively, d is the spacing vector, $x_{n}$ is the element position, and k is the wavenumber.

It should be clarified that Equation (5) is the differentiable reformulation of Equation (1) for integration into the PI-GAN framework. While Equation (1) expresses the total array pattern as the superposition of individual AEPs weighted by complex excitations in a general form, Equation (5) explicitly decomposes the complex excitation into amplitude and phase components and incorporates the spatial phase term *e*^jkx·cosθ^, making the forward electromagnetic computation fully differentiable. The AEP of each element is predicted by the pretrained surrogate DNN rather than obtained from full-wave simulation, enabling gradient backpropagation through the physics layer during GAN training.

The discriminator D evaluates whether the input radiation pattern originates from the target distribution or the generator:

(6) $$D:D(y;\theta_{D})\to [0,1].$$

The training process follows a minimax game formulation. The discriminator loss employs binary cross-entropy:

(7) $$L_{D}=-E_{y\sim p_{\mathrm{data}}}[\log D(y)]-E_{z\sim p_{z}}[\log(1-D(P(G(z))))].$$

In the discriminator loss, p_data_ represents the distribution of real radiation pattern samples used for adversarial training. In this work, the real data distribution p_data_ consists of ideal Chebyshev array patterns generated with the target sidelobe level, which serve as the reference for high-quality radiation patterns.

The generator loss comprises an adversarial component and a physics-based pattern matching component:

(8) $$L_{\mathrm{adv}}=-E_{z\sim p_{z}}[\log D(P(G(z)))],$$

(9) $$L_{\mathrm{phy}}=E_{z\sim p_{z}}\left[{\left\|P(G(z))-y_{\mathrm{target}}\right\|}_{2}^{2}\right],\;\mathrm{and}$$

(10) $$\min_{\theta_{G}}L_{G}=\lambda_{\mathrm{adv}}L_{\mathrm{adv}}+\lambda_{\mathrm{phy}}L_{\mathrm{phy}},$$

where y_target_ in the physics-based loss function denotes the target radiation pattern that the generated array should approximate, which is the same Chebyshev pattern used as the real data. The weighting coefficients are set to λ_adv_ = 0.05 and λ_phy_ = 100, respectively. The rationale for this configuration is that the physics-based loss, which directly measures the discrepancy between the generated and target radiation patterns, should dominate the optimization to ensure that the synthesized array meets the physical design specifications. The adversarial loss serves as a regularization term that encourages the generator to explore diverse solutions and avoid mode collapse, but its weight is kept relatively small to prevent training instability. This weight configuration effectively balances adversarial training and physical target matching, as demonstrated by the stable convergence behavior observed in the experiments.

The PI-GAN framework is trained using the Adam optimizer with a learning rate of 0.001 for the generator and 0.0001 for the discriminator. The batch size is set to 64, and the training proceeds for 8000 epochs. A step learning rate scheduler is employed, halving the learning rate every 1000 epochs to facilitate fine-grained convergence. The latent vector dimension is 32, sampled from a standard normal distribution. All experiments are conducted on a personal computer equipped with an Intel Core i5-12600KF CPU (3.70 GHz), 32 GB RAM and an NVIDIA GeForce RTX 4060 Ti GPU. It should be clarified that the PI-GAN optimization time does not include the AEP surrogate model pretraining. Once the AEP surrogate model is pretrained, it can be reused for different target patterns without retraining.

## 3. Experiment Results and Discussion

The effectiveness of the proposed PI-GAN method is validated through comprehensive synthesis experiments on nonuniform arrays of various scales.

### 3.1. 16-Element Array Synthesis

The first experiment focuses on sparse array optimization for a 16-element linear array’s spacings and excitations. The antenna element is the microstrip patch operating at 3 GHz described in Section 2.1. The objective is to minimize the peak sidelobe level (PSLL) by optimizing element spacings within [0.5λ, λ]. The proposed PI-GAN method is compared with particle swarm optimization (PSO) combined with the same AEP surrogate model.

For fair comparison, the PSO algorithm is combined with the same AEP surrogate model to evaluate the radiation pattern, ensuring that both methods are evaluated under identical mutual coupling conditions. The PSO is configured with 60 particles and 600 iterations. The inertia weight decreases linearly from 0.9 to 0.4, and the learning factors are set to c1 = c2 = 2.0. The PSO algorithm searches for optimizing a 16-element linear array within the same constraints as the PI-GAN framework. The total optimization time of PSO is 563 s. In contrast, the PI-GAN method requires only 62 s, achieving an efficiency improvement of approximately 89%.

In addition to the comparison with PSO, the proposed PI-GAN framework is also compared with a machine-learning-based array synthesis method to demonstrate its advantages and competitiveness. Physics-aware generative adversarial network (PAGAN) [20] was also a synthesized 16-element linear array. The PAGAN framework adopts a GAN architecture similar to the proposed PI-GAN architecture, but it embeds the idealized array factor formula directly into the generator loss function as the physics constraint. The generator network consists of three fully connected layers (input: 32-dim latent vector, hidden: 128 and 256 neurons with LeakyReLU activation, and output: 3N-1 parameters for spacings, amplitudes, and phases). The discriminator network also consists of three fully connected layers. The array optimal solutions can be obtained faster by using the PAGAN method, but training takes approximately 8 h. The key limitation of PAGAN is that it uses the idealized array factor model, which assumes isotropic radiating elements and neglects mutual coupling effects between elements. This may lead to inaccurate predictions for closely spaced nonuniform arrays where mutual coupling significantly alters the radiation characteristics.

**Figure 5:**
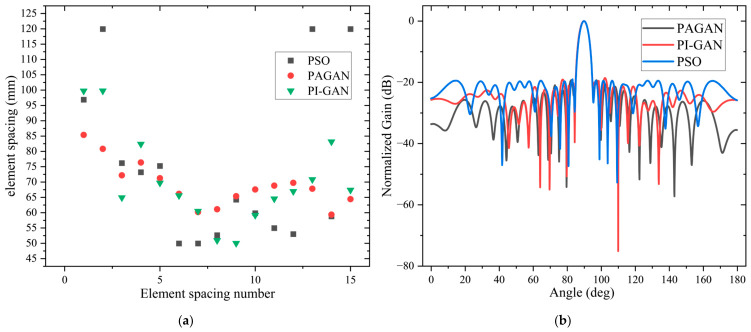
Optimization results of three methods for the 16-element linear array methods: (**a**) element spacing distributions for all three methods, and (**b**) radiation patterns of PAGAN, PSO, and PI-GAN.

Figure 5a shows the optimized element spacing distributions obtained by three methods, and Figure 5b presents the corresponding radiation patterns.

Table 1 summarizes the quantitative comparison between the three methods. The PI-GAN approach demonstrates superior sidelobe suppression and dramatically reduced computation time.

**Figure 6:**
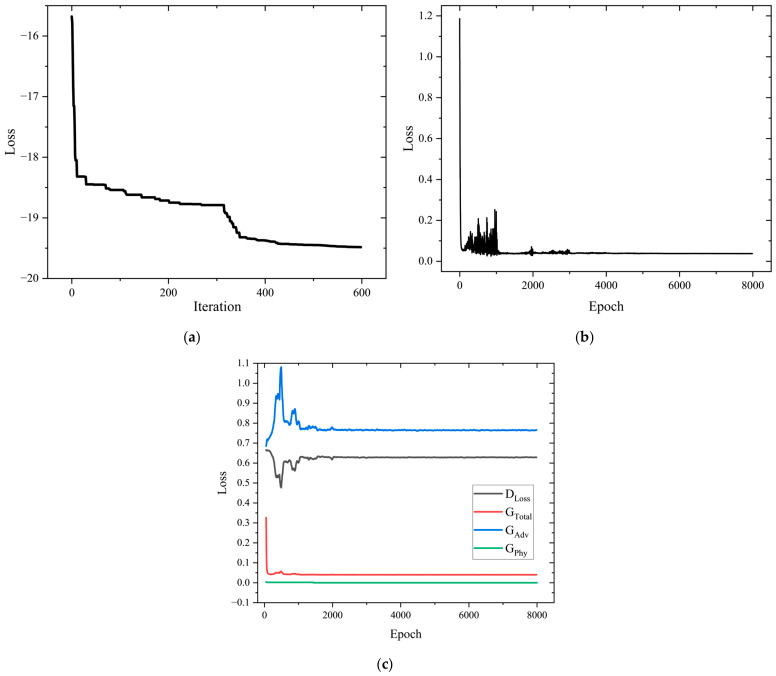
Training convergence curves of three methods for the 16-element linear array: (**a**) PSO, (**b**) PAGAN, and (**c**) PI-GAN.

To demonstrate the training stability and convergence behavior of the proposed PI-GAN framework, the training convergence curves of three methods for the 16-element linear array are recorded over 8000 epochs, as shown in Figure 6. The discriminator loss stabilizes at approximately 0.62–0.63 after approximately 2000 epochs, indicating that the discriminator reaches equilibrium with the generator. The curves in Figure 6c display the unweighted adversarial loss L_adv_ (labeled as “G_Adv_”), the unweighted physics-based loss L_phy_ (labeled as “G_phy_”), and the weighted total generator loss L_G_ = λ_adv_ L_adv_ + λ_phy_ L_phy_ (labeled as “Gtotal”), respectively. At the early training stage (epoch ≈ 100), L_adv_ ≈ 0.63, L_phy_ ≈ 0.0104, and the weighted physics loss 100 L_phy_ ≈ 1.04 dominates L_G_ ≈ 1.08, confirming that the physics-based constraint drives the initial pattern matching. As training proceeds, L_phy_ decreases rapidly to approximately 1^−6^ (weighted contribution ≈ 1^−4^), while L_adv_ stabilizes at approximately 0.75–0.77, suggesting that the generator successfully fools the discriminator while maintaining physical consistency. At convergence, the total generator loss L_G_ ≈ 0.038, satisfying L_G_ = 0.05 L_adv_ + 100 L_phy_ as defined in Equation (10).

### 3.2. Three-Hundren-Element Planar Array Synthesis

**Figure 7:**
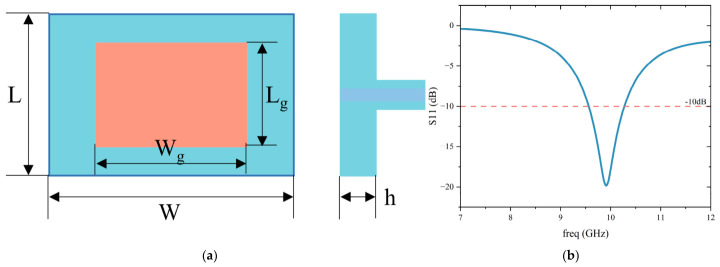
Geometry of the 10 GHz microstrip patch antenna element for the planar array: (**a**) unit model, and (**b**) $S_{11}$ curve.

To demonstrate the effectiveness of the PI-GAN approach for planar arrays, a 300-element nonuniform planar array is synthesized. The antenna element is the microstrip patch operating at 10 GHz described in Figure 7. The structural parameters of the antenna are shown in Table 2. The optimization variables are the (x,y) coordinates and complex excitations of all elements. The target is to achieve dual principal-plane Chebyshev patterns with −25 dB sidelobe levels in both ϕ=0° and ϕ=90° cuts. The planar AEP prediction model is adaptively constructed based on an effective coupling radius and the number of neighboring elements within this region.

**Figure 8:**
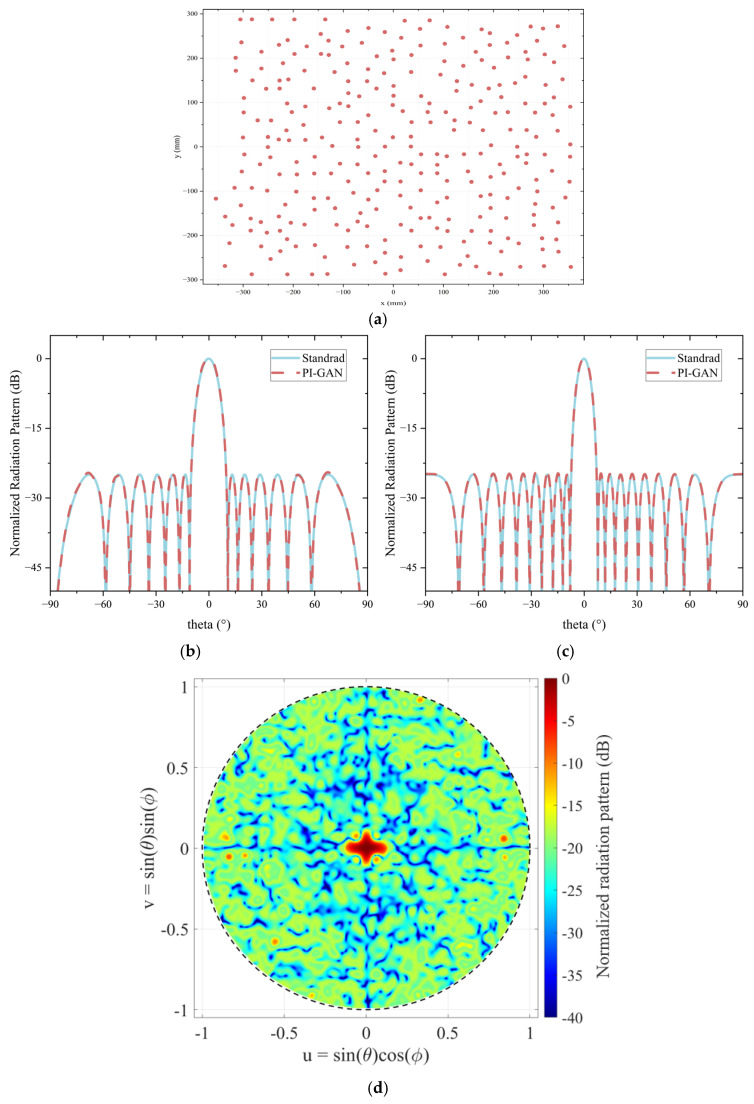
PI-GAN optimized planar array results: (**a**) element distribution, (**b**) phi = 0 tangent plane radiation pattern, (**c**) phi = 90 tangent plane radiation pattern, and (**d**) 3D radiation pattern.

Figure 8 shows the optimized element layout and the 3D radiation pattern from the full-wave simulation, verifying that the −25 dB PSLL specification is met in both principal planes.

For comparison, the PSO algorithm is also applied to the 300-element planar array synthesis under the same AEP surrogate model and optimization constraints. Despite extensive parameter tuning, the PSO algorithm fails to meet the −25 dB target, achieving a PSLL of only −20.72 dB. This significant performance gap demonstrates the limitation of the PSO algorithm in handling high-dimensional, non-convex array synthesis problems, and it further highlights the advantage of the proposed PI-GAN framework in achieving superior performance for large-scale planar arrays.

### 3.3. Experimental Validation

**Figure 9:**
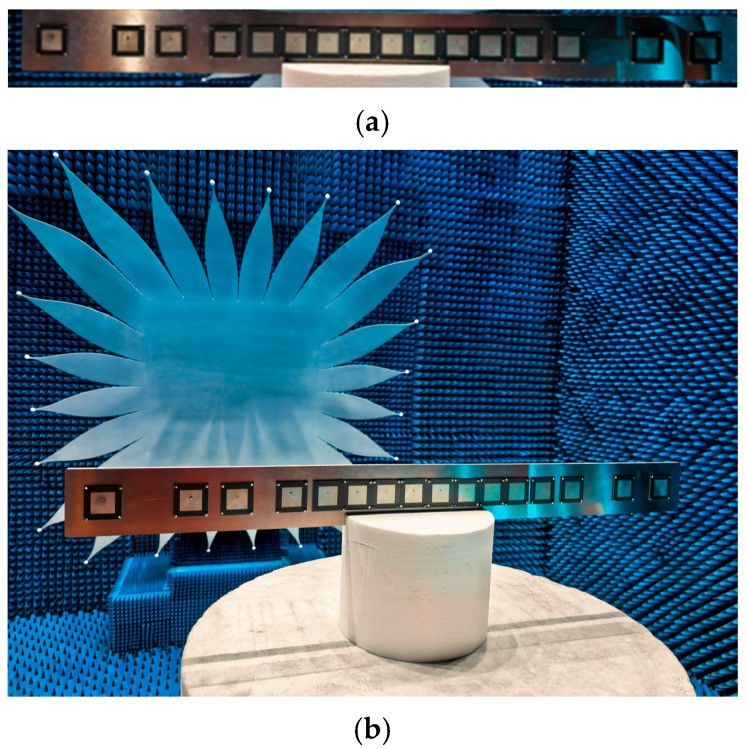
Photograph and measurement setup of the fabricated 16-element linear array: (**a**) array prototype, and (**b**) anechoic chamber measurement setup.

To further validate the practical feasibility of the PI-GAN synthesized array, a 16-element nonuniform linear array prototype is fabricated and measured. The physical implementation of the array and the far-field measurement setup in an anechoic chamber are shown in Figure 9a and Figure 9b, respectively. The array elements are rectangular microstrip patches operating at 3 GHz, and the optimized nonuniform spacings obtained from the PI-GAN synthesis are precisely implemented on a Rogers RO4350B substrate.

**Figure 10:**
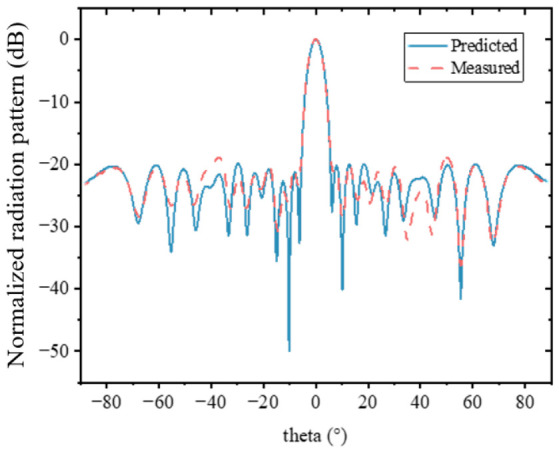
Radiation pattern test results of the fabricated 16-element array.

Figure 10 compares the measured and simulated radiation patterns of the fabricated prototype in the broadside direction. Excellent agreement is observed between the two curves, with the measured PSLL reaching −18.97 dB, which is only 0.51 dB higher than the full-wave simulated value of −19.48 dB. The slight discrepancies between the simulated and measured results mainly stem from manufacturing deviations, connector inconsistencies, intrinsic approximation errors of the AEP surrogate model, and residual reflections in the measurement environment. These experimental results convincingly demonstrate that the proposed PI-GAN framework is capable of generating array configurations that not only perform well in simulation but also maintain high fidelity in physical realization, confirming its practical engineering value for realistic antenna array design.

## 4. Conclusions

This paper presents a PI-GAN framework for the efficient and physically consistent synthesis of nonuniform antenna arrays with mutual coupling effects. The key advantages of the proposed method are its use of GAN for powerful global optimization, accurate consideration of mutual coupling via an embedded differentiable AEP surrogate, and demonstrated effectiveness for both linear and planar arrays. Experiments on a 16-element linear array and a 300-element planar array show that PI-GAN outperforms PSO in sidelobe suppression and computational efficiency. A fabricated prototype of the 16-element array is measured, with the experimental results closely matching the simulations, confirming its practical feasibility.

The current framework has several limitations that should be acknowledged. First, the AEP surrogate model is trained for a specific antenna element type and operating frequency, and retraining is required when different antenna elements or frequencies are used. Second, the proposed method has been validated for linear and planar arrays with specific element types, and its applicability to conformal arrays and arrays with more complex element structures requires further investigation. Third, the current framework assumes narrowband operation, and extension to wideband arrays necessitates additional frequency-dependent AEP modeling. Regarding generalization capability, the subarray-based AEP prediction model exhibits good scalability to different array sizes due to the transfer learning property, as the same pretrained model can be applied to arrays of different scales. Future work will focus on extending the method to wideband arrays, conformal arrays, and dynamic beam-scanning arrays with real-time constraints.

## Figures and Tables

**Table 1:** Comparison of the 16-element linear array optimization results.

Method	Network Architecture	Physics Model	PSLL (dB)	Time (s)	Training Data
PAGAN	3-layer GAN (128–256)	Array Factor	−19.34	41	Unsupervised
PSO	N/A (Heuristic)	AEP Surrogate	−19.05	563	N/A
PI-GAN (Ours)	3-layer GAN (128–256)	AEP Surrogate	−19.48	62	Unsupervised

**Table 2:** Structural parameters of the microstrip patch antenna.

Variable	Value	Variable	Value
L	11.8585 mm	W_g_	11.8585 mm
W	13.3427 mm	h	1.575 mm
L_g_	9.0707 mm	$\varepsilon_{r}$	2.2

## Data Availability

All data generated or analyzed during this study are included in this manuscript. There are no additional data or datasets beyond what is presented in the manuscript.
